# Extended-sleeve resection for endoluminal recurrence of B3 thymoma: does aerogenous spread really exist?

**DOI:** 10.1093/icvts/ivae105

**Published:** 2024-05-28

**Authors:** Simone Tombelli, Stefano Bongiolatti, Valeria Pasini, Luca Voltolini

**Affiliations:** Division of Thoracic Surgery, Careggi University Hospital, Florence, Italy; Division of Thoracic Surgery, Careggi University Hospital, Florence, Italy; Section of Anatomic Pathology, Department of Health Science, University of Florence, Florence, Italy; Division of Thoracic Surgery, Careggi University Hospital, Florence, Italy

**Keywords:** Thymoma, Aerogenuos spread, Extended**-**sleeve lobectomy, Thymectomy

## Abstract

Thymomas are a variant of thymic epithelial tumours. They are considered malignant due to their tendency to local invasion and they showed lower metastatic behaviour. Distal metastasis is rare and an endobronchial mass is a rare presentation. First-line treatment for early-stage thymomas is surgery; for Masaoka–Koga stage III, neoadjuvant or adjuvant chemoradiation therapy should be considered in association with surgery after Multidisciplinary Tumour Board evaluation. We report a rare case of radical resection with type A extended-sleeve lobectomy in a 63-year-old woman who was affected by endobronchial recurrence of B3 thymoma, 31 months after complete and radical thymectomy.

## INTRODUCTION

Thymomas are malignant tumours with a tendency to local invasion. They may exhibit more aggressive behaviour, including invasion of adjacent organs or distant metastases. Neoadjuvant treatment is recommended for direct involvement of the mediastinal structures precluding an upfront radical surgery The most common sites for intrathoracic recurrence are contiguous organs such as the mediastinum, pleura and lungs. Direct airway involvement is extremely rare [1, 2].

## CASE DESCRIPTION

**Figure 1:**
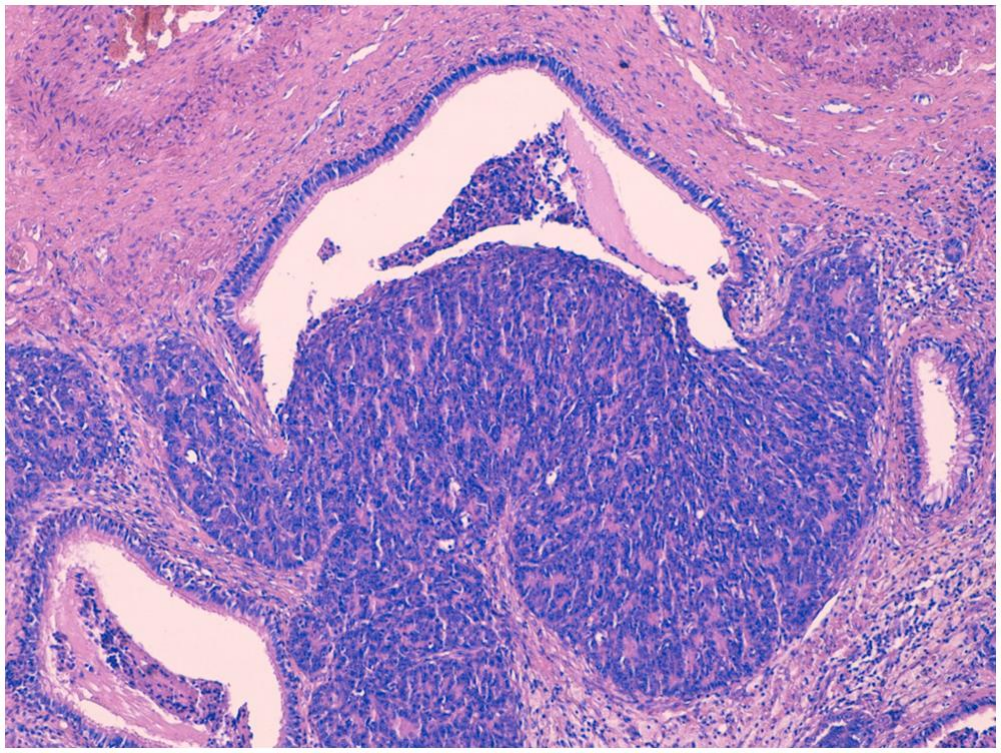
Type B3 thymoma infiltrating a bronchiole (hematoxylin and eosin, 10×). The tumour is composed of polygonal cells with mild nuclear atypia and inconspicuous nulceoli.

We present the clinical case of a 63-year-old female patient affected by locally-advanced B3 thymoma. The lesion was 41 mm × 50 mm in size and surrounded the right upper lobar bronchus with endobronchial extension of hypodense material; the mass encircled the superior lobar pulmonary artery (Video 1). She was treated with a multimodal approach starting with neoadjuvant chemotherapy (Cyclophosphamide, Cisplatin, Doxorubicin). The restaging computed tomography (CT) scan showed a partial response to the neoadjuvant treatment with a minimum reduction of the lesion’s size (41 mm × 47 mm). A complete radical thymectomy associated with wedge resection of the right upper lobe (with negative margins at the frozen section) through sternotomy was performed. The final pathology revealed a type B3 Masaoka–Koga stage III thymoma (ypT3 Nx) with free margins. Within the pulmonary localization an area of neoplastic cells invading through a bronchiole wall was found (Fig. 1). The postoperative course was uneventful and the patient started adjuvant radiotherapy (50 Gy) and regular follow-up CT scan evaluation.

Thirty-one months after surgery, the patient was admitted to the emergency department for loss of consciousness. The contrast-enhancement CT scan showed an occluding endoluminal lesion of the right main bronchus protruding into the trachea (Video 1). The following day, a rigid bronchoscopy was performed with laser-assisted removal of the endobronchial part of the lesion and restoration of the patency of the right main bronchus. Biopsy was consistent with B3 Thymoma recurrence. She then underwent a placement of a Dumon silicone prosthesis covering the right upper lobar bronchus to prevent the regrowth of the lesion in the bronchial tree.

**Figure 2:**
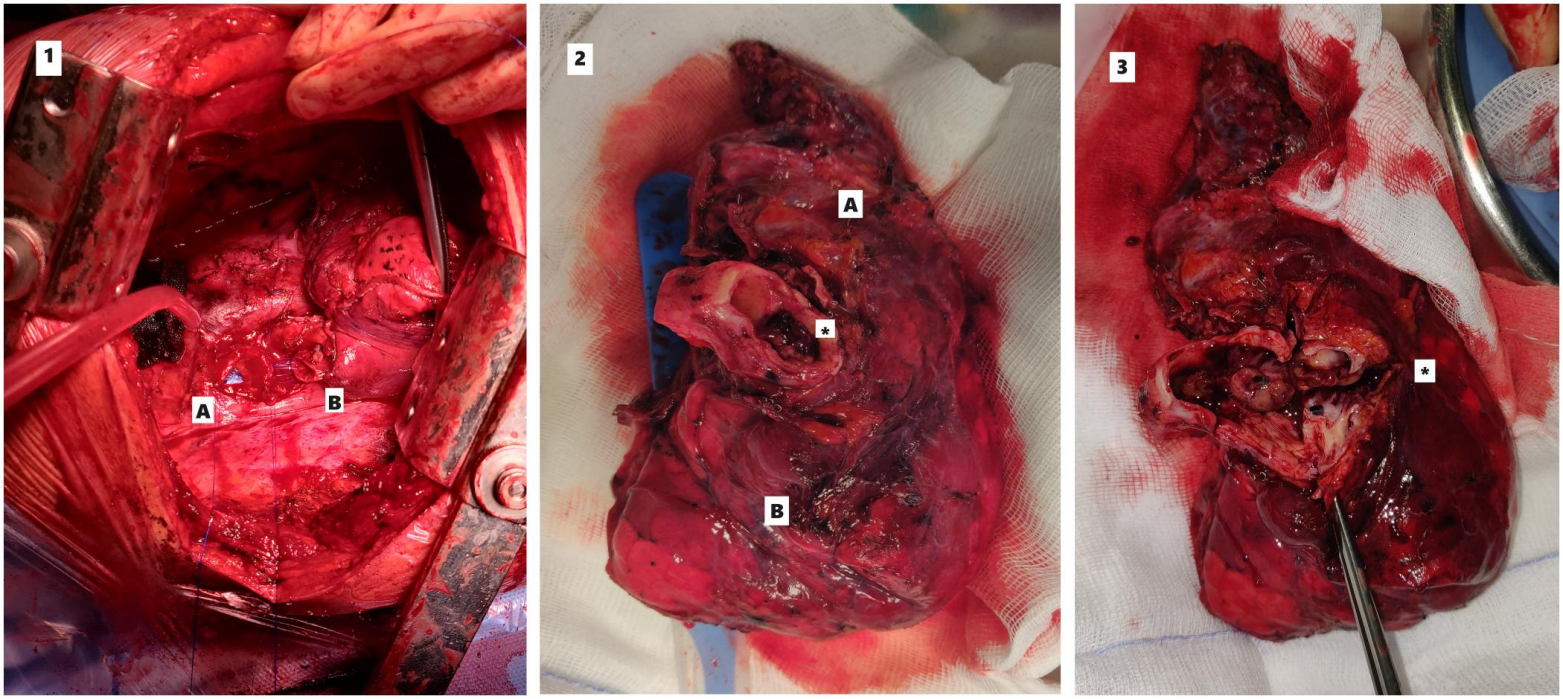
(1) Suture between right main bronchus (A) and right inferior bronchus (B). (2) Surgical specimen: (A) right upper lobe and (B) middle lobe. (3) The tumour is visible (*) and occludes the entire lumen of the Right Upper Bronchus involving the Intermediate Bronchus.

After a new multidisciplinary tumor board evaluation, radical surgery was considered appropriate for this local recurrence and a type A extended-sleeve lobectomy was performed via posterolateral thoracotomy [3] due to the invasion of the right upper lobe and the proximal part of the bronchus intermedius close to the origin of the middle lobe bronchus (Fig. 2). Seven days after surgery, the patient was discharged home. The bronchoscopy showed good healing of the bronchial anastomosis. The pathologic specimen appears to be consistent with B3 Thymoma. Once again, R0 resection was achieved. The 1-year follow-up CT scan showed no local recurrence and the patient was in good general condition (Video 1).

## DISCUSSION

Surgery is the gold standard for the treatment of early-stage Thymoma. For more advanced cases (Masaoka–Koga stage III and super-selected stage IVA) surgery can be considered a first-line therapy if complete resection is achievable [4]. For patients with locally advanced, marginally resectable, thymoma the use of neoadjuvant therapy should be considered and discussed at an MDT. After histologic assessment, therapeutic options include neoadjuvant chemotherapy or concurrent chemo-radiotherapy [4]. Radiotherapy is usually accepted as adjuvant treatment when complete R0 resection is not achieved or when adjacent organs are involved.

To our knowledge, only 21 cases of endobronchial polypoid growth have been described in the literature [1, 2]. Direct airway involvement is extremely rare and the mechanism of this development remains controversial. Honda *et al.* [5] proposed a mechanism of spread from the adjacent pleura leading to parenchymal involvement, with invasion of the distal bronchial wall, resulting in the growth of a polypoid endobronchial tumour. However, in our specimen, no neoplastic proliferation in the first parenchymal and bronchial resection margins and lymph nodes was detected, suggesting a different mechanism of spread from that proposed by Honda. In our case, we then would exclude contiguous spread of the lesion from the previous wedge resection based on the pathological findings. In the first surgical specimen, although the margins were clear, an area of neoplastic cells was found within the lung localization that had invaded through a bronchiolar wall. We therefore hypothesize that this could be an exceedingly rare case of aerogenous spread and bronchial implantation of a completely resected B3 thymoma treated with neoadjuvant chemotherapy and adjuvant radiotherapy.

## Data Availability

All relevant data are within the manuscript and its Supporting Information Files.
